# Progress in research on paclitaxel and tumor immunotherapy

**DOI:** 10.1186/s11658-019-0164-y

**Published:** 2019-06-13

**Authors:** Linyan Zhu, Liqun Chen

**Affiliations:** College of Biological Science and Engineering, Fuzhou University, Fuzhou, 350108 China

**Keywords:** Paclitaxel, Anticancer mechanism, Endophytic fungus, Biosynthetic pathway

## Abstract

Paclitaxel is a well-known anticancer agent with a unique mechanism of action. It is considered to be one of the most successful natural anticancer drugs available. This study summarizes the recent advances in our understanding of the sources, the anticancer mechanism, and the biosynthetic pathway of paclitaxel. With the advancement of biotechnology, improvements in endophytic fungal strains, and the use of recombination techniques and microbial fermentation engineering, the yield of extracted paclitaxel has increased significantly. Recently, paclitaxel has been found to play a large role in tumor immunity, and it has a great potential for use in many cancer treatments.

## Introduction

**Fig. 1:**
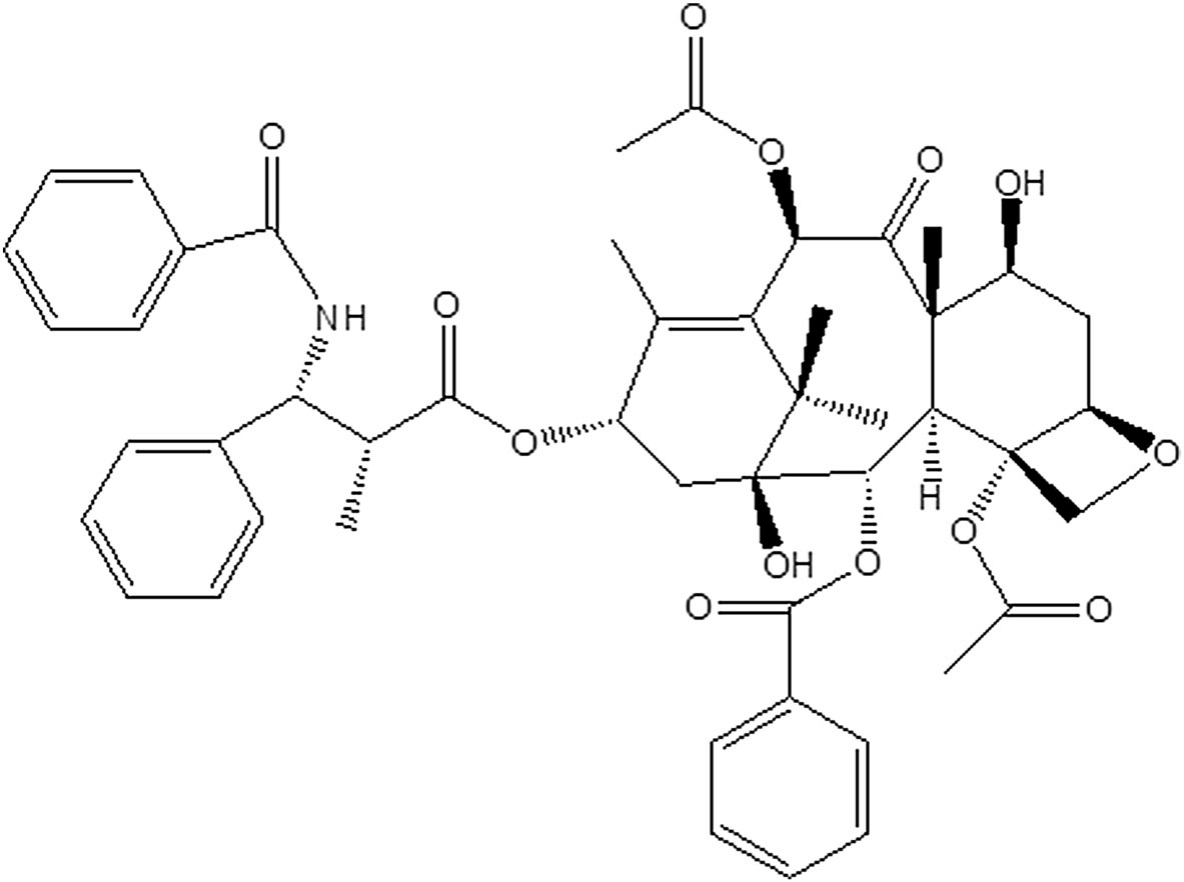
Chemical structure of paclitaxel

Paclitaxel (trade name Taxol) is a tricyclic diterpenoid compound naturally produced in the bark and needles of *Taxus brevifolia*. Its molecular formula is C_47_H_51_NO_14_, and its chemical structure is shown in Fig. 1. Because of its unique anticancer mechanism, it is already one of the most successful and widely used natural anticancer drugs [1]. Unlike other tubulin-binding anticancer drugs, which prevent the assembly of tubulin into microtubules, paclitaxel promotes the assembly of tubulin into microtubules and prevents the dissociation of microtubules, blocking cell cycle progression, preventing mitosis, and inhibiting the growth of cancer cells [2]. It is also used in coronary heart disease, skin disorders, renal and hepatic fibrosis, inflammation, and axon regeneration, and clinical trials are being conducted for degenerative brain diseases [3].

After a series of clinical trials, the US Food and Drug Administration (FDA) approved paclitaxel for the treatment of advanced ovarian cancer in 1992 [4]. Since then, paclitaxel has been widely used in the treatment of breast cancer, colorectal cancer, and squamous cell carcinoma of urinary bladder. Furthermore, it has been used in the treatment of diseases such as head and neck cancers, small-cell and non-small-cell lung cancers (NSCLCs), and AIDS [5].

Paclitaxel was originally isolated from *T. brevifolia*. However, due to the relatively low concentration of paclitaxel present in the plant, paclitaxel is extremely expensive [6, 7]. Thus, several other ways to obtain paclitaxel have been developed, including the artificial cultivation of *T. brevifolia*, chemical synthesis or semi-synthesis of the drug, and biotechnological synthesis. The extraction of paclitaxel from genetically modified endophytic fungi, in particular, has proven to be an effective way to obtain the drug.

### Anticancer mechanism of paclitaxel

In 1979, it was reported that paclitaxel promotes the assembly of microtubules, structures that consist of repeating subunits composed of α/β-tubulin heterodimers. Paclitaxel reduces the critical concentration of assembled tubulin subunits and increases the percentage of assembled tubulin subunits [2]. During the prophase, microtubules form a spindle to pull the chromosomes towards the poles. During later stages, they depolymerize and the spindle structure dissolves. Both exposure to cold temperatures and exposure to calcium ions trigger the depolymerization of microtubules. Paclitaxel binds to and stabilizes microtubules, and paclitaxel-bound microtubules resist depolymerization, even upon treatment under cold temperatures or with calcium ions. Therefore, paclitaxel treatment promotes tubulin polymerization and blocks the progression of mitosis [8, 9].

### Paclitaxel source

#### Artificial cultivation of *Taxus* plants

Large amounts of paclitaxel are used in both clinical and basic research. However, some *Taxus* species are nearly paclitaxel-deficient, and excessive utilization of these plants could destroy the natural ecological balance in which they exist. Artificial cultivation methods have been used to reduce the effects on the ecosystems. Moreover, a large effort has been made in the Yunnan and Sichuan provinces in China to plant *Taxus chinensis*, which has been identified as a useful source of the drug. To date, there are more than 150 *T. chinensis* plantations. These guarantee a stable plant population, from which several active pharmaceutical ingredients, including paclitaxel, can be extracted [10]. This is one of the most effective means by which to obtain paclitaxel. However, over-reliance on epigenetic and environmental factors and the slow growth rate of *Taxus* plants are issues that are still a cause of concern.

### Chemical synthesis of paclitaxel

#### Chemical synthesis

In 1994, Holton and coworkers successfully synthesized paclitaxel [11]. However, paclitaxel has a complex molecular structure, and its synthesis is very complicated; the process entails a total of 25–40 steps. Moreover, the commercial production of paclitaxel is not yet feasible, as the reaction conditions are extremely difficult to control, large amounts of toxic products are produced, and the cost of production is extremely high.

### Chemical semi-synthesis

In 1988, the first use of chemical semi-synthesis to synthesize paclitaxel from 10-deacetylbaccatin III (10-DAB) was reported [12]. Paclitaxel is produced through chemical semi-synthesis by converting analogs or precursors found in *Taxus* plants, such as 10-deacetylbaccatin and baccatin III, into paclitaxel. This can be done through the asymmetric epoxidation pathway, asymmetric double hydroxylation reaction, chiral auxiliary strategy, Diels–Alder reaction, or enol imine condensation, among other methods [13, 14]. Asymmetric hydroxylation is a chemical reaction in which an olefin is converted into a vicinal diol in the presence of ruthenium tetroxide with a chiral quinine ligand. This reaction typically requires a catalytic amount of citric acid with potassium ferricyanide or N-methylmorpholine as well as an N-oxide regeneration reaction. This method both reduces the level of toxic emissions produced during paclitaxel synthesis and lowers the price.

### *Taxus* tissue culture

The amounts of paclitaxel found in different parts of *Taxus* plants, organs, and tissues greatly differ; therefore, explants of *Taxus* have been cultured in a targeted manner. In 1989, *T. brevifolia* was first cultured successfully. After 2 years, *T. brevifolia* cultures yielded 1–3 mg/l of paclitaxel per extraction [15]. Since then, many other *Taxus* species have been found to grow well, including *T. baccata*, *T. yunnanensis*, *T. cuspidate*, *T. chinensis*, *T. canadensis*, and *T. globosa*. Breeding with high-yield cell lines, using two-stage culture systems, optimizing carbohydrate sources, using pre-feeding strategies, and using fungal culture inducers (e.g., fungal extracts plus salicylic acid, vanadyl sulfate, chitosan, squalene, and methyl jasmonate) are all effective means by which to increase the yield of paclitaxel [16, 17].

*Taxus* cell culture has many advantages. Cultures, unlike wild plants, are unaffected by weather, seasons, and environmental pollution. Culturing *Taxus* cells also allows for the continuous production of paclitaxel of identical purity, and the cultures are renewable, environmentally friendly, and source-independent. Culturing *Taxus* cells is a good way to obtain paclitaxel, especially in combination with metabolic and genetic engineering techniques that increase the yield. However, there are several difficulties associated with culturing *Taxus* cells. Culturing can be hindered by slow cell growth, stress factor generation, induction difficulties, cell aggregation, increased cellular shear sensitivity, high costs, and unstable yields. Innately undifferentiated cambial meristematic cells (CMCs) circumvent many of the problems associated with traditional dedifferentiated cells (DDCs). To bypass the dedifferentiation step, Lee isolated and cultured CMCs which produced a combined total of 264 mg of paclitaxel per kg of cells and 74% of this was secreted directly into the medium [18, 19]. These cells may provide a cost-effective and environmentally friendly platform for the sustainable production of a variety of important natural plant products.

### Production of paclitaxel using endophytic fungi

#### Study on paclitaxel production by endophytic fungi

In 1993, Stierle and Strobel isolated *Taxomyces andreanae*, an endophytic fungus, from *T. chinensis* and confirmed by mass spectrometry, chromatography, and immunochemistry that it produced paclitaxel. They extracted paclitaxel under artificial culture conditions in vitro, but the yield was very low (24–50 μg/l) [20]. In 2003, Chen also isolated strains of endophytic fungi from the inner bark and branches of *T. yunnanensis*. By thin-layer chromatography and high-performance liquid chromatography analysis of 52 strains of endophytic fungi, it was found that 19 strains can produce taxol and taxane [21]. Endophytic fungal biosynthesis technology has opened a new branch of the global paclitaxel market, which is considered to be worth billions of dollars, when the concentration of paclitaxel extracted from endophytic fungi increased to 846 μg/l [22].

There is some evidence that suggests that endophytic fungi can synthesize paclitaxel or paclitaxel analogs and that the microbial synthesis pathway of paclitaxel in endophytic fungi is significantly different from the biosynthesis pathway of paclitaxel in *Taxus*. Due to its complexity, the microbial synthesis pathway remains unclear, and some steps in this pathway are different from the known synthetic pathway. Heining and coworkers found that it was not possible to demonstrate the independent synthesis of taxanes in any endophytic fungus, including the first published endophytic fungus, *T. andreanae* [23]. Kusari and coworkers stated that further research on the production of paclitaxel using endophytic fungus biotechnology is needed [24]. Due to this, the use of endophytic fungi to produce paclitaxel remains a controversial issue.

### Methods to increase the production of paclitaxel by endophytic fungi

The genetic properties of endophytic fungi can be altered by physical, chemical, or aerospace techniques, as well as by complex mutagenesis. Protoplast fusion is a technology by which plant cells with distant phylogenetic relationships are integrated, and this technique can be used to achieve long-range hybridization of cells and expand the recombination range of genetic material. It is a powerful tool in the modification of genetic material, and, as such, it plays a significant role in microbial genetic breeding. Due to their non-polar membranes, microbial cell protoplasts are more easily fused, allowing the whole cytoplasms and nuclei to fuse with each other while leaving the genetic material intact, allowing for the production of hybrids. The combination of mutational and protoplast fusion techniques is effective in increasing the amount of paclitaxel produced by endophytic fungi [25].

The biotechnological screening of high-producing fungal strains is widely used to enhance the yield of filamentous fungi. It is a valuable tool in the identification of useful genetic material and suitable transformation methods for fungi—particularly endophytic fungi. Advances in DNA recombination technology have resulted in updates to the original metabolic pathway scheme, and the successful expression of the microbial paclitaxel synthesis pathway genes was a breakthrough in improving yield of paclitaxel. The identification and cloning of key enzyme genes in the microbial paclitaxel synthesis pathway, determination of suitable vectors, and genetic engineering techniques to study the expression of exogenous genes have all contributed to increasing the yield of paclitaxel produced by endophytic fungi.

### Biosynthesis pathway of paclitaxel

**Fig. 2:**
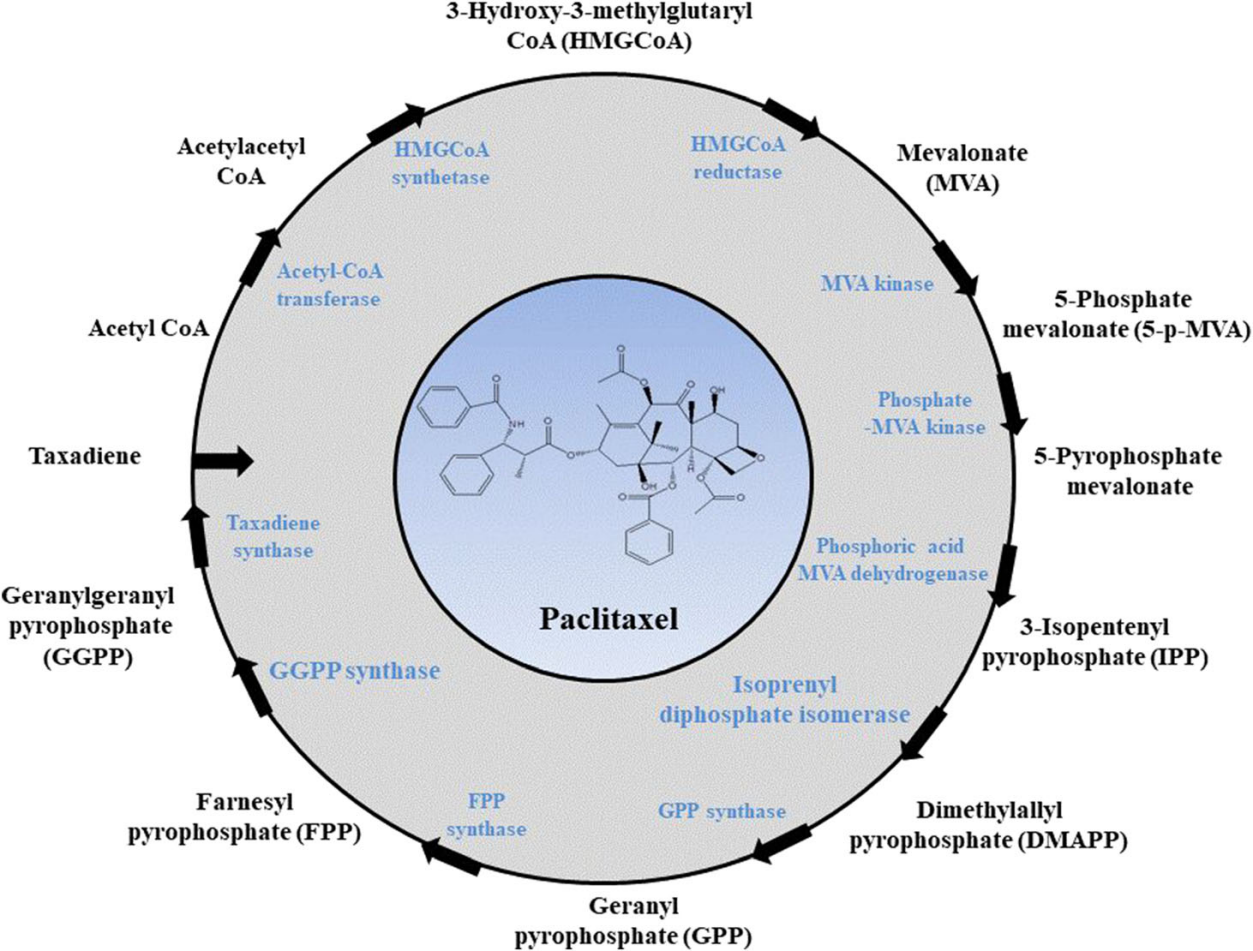
The biosynthetic pathway of paclitaxel

The biosynthetic pathway of paclitaxel includes at least 19 steps. In recent years, scientists have made significant progress in characterizing each step [16]. The biosynthesis of paclitaxel involves the reaction between isopentenyl pyrophosphate (IPP) units and dimethylallyl pyrophosphate (DMAPP), which are obtained from the mevalonate (MVA) pathway and the 2-C-methyl-erythritol-4-phosphate (MEP) pathway, respectively [26]. In the presence of taxadiene synthase (TS), geranylgeranyl pyrophosphate (GGPP) is cyclized to form taxa-4(5),11(12)-diene. The biosynthetic pathway involves enzymes from several different classes that are located in several different cellular compartments, including the plastid, endoplasmic reticulum and cytosol. Modifications, such as acylation and ketonation of baccatin, are carried out. Then, the functional side chain groups are added, ending in the complete synthesis of paclitaxel [27]. The synthesis pathway is summarized in Fig. 2.

Part of the biosynthetic pathway of paclitaxel has been transferred to heterologous expression systems, such as *Saccharomyces cerevisiae*, *Escherichia coli*, and certain plants. IPP isomerase, GGPP synthase, and TS have been overexpressed in *E. coli* to synthesize taxadiene, and the production level of taxadiene from cultured cells has been recorded to be as high as 1.3 mg/l [28]. In *S. cerevisiae*, an early pathway from primary metabolism to taxol-5α-acetoxy-10β-alcohol was established. Now, a multivariate modular approach in *E. coli* is used to obtain the highest paclitaxel yields. This approach divides the metabolic pathway of paclitaxel into two modules; in the first, IPP is formed from heterogeneous upstream MEP, and in the second, terpenoids are formed. The highest recorded concentration of paclitaxel extracted from *E. coli* was 570 mg/l, and this was achieved by optimizing the P450 expression of taxanes, combining different cross-reductases, and modifying the N-termini of different enzymes [29].

Many enzymes participate in the biosynthetic pathway. Genetic and metabolic engineering can be used to produce these enzymes and obtain higher yields of paclitaxel at the cellular and molecular levels. Due to its complexity, this pathway is not well understood, and further research is needed to fully characterize and describe it.

### Paclitaxel and tumor immunotherapy

#### Tumor immunotherapy

Tumor immunotherapy produces an immune response to tumors by improving the body’s immunity. Recently, advances have been made in our understanding of the possible applications of classic drugs in tumor immunotherapy. Many studies have shown that paclitaxel directly kills tumor cells and regulates various immune cells, such as effector T cells, dendritic cells (DCs), natural killer (NK) cells, regulatory T cells (Tregs), and macrophages [30]. Other chemotherapeutics have similar immunomodulatory properties, such as belinostat [31], doxorubicin [32], bleomycin [33], and bortezomib [34]. Tumor immunotherapy works through several mechanisms: (1) by promoting the proliferation and activation of T cells; (2) by increasing B-cell activity and increasing antibody production; (3) by increasing the number of NK cells, the production of active substances, and the ability to present antigens; and (4) by improving the body’s hematopoietic function.

### Paclitaxel and immune cells

Carboplatin and paclitaxel (CP) chemotherapy is used as a second-line chemotherapy regimen, and it is commonly used to treat melanoma. Carboplatin down-regulates the T-cell inhibitory molecule programmed death receptor-ligand 2 (PD-L2), which is expressed by DCs and melanoma cells to enhance T-cell activation [35]. In addition, paclitaxel reduces the number of Tregs, aids in the production of the cytokine interleukin-10 (IL-10), transforms growth factor-beta by Tregs, and stimulates DC-mediated antigen presentation. A study has shown that the peptide-pulsed DC vaccine in combination with CP therapy (DCCP) is more likely to be effective than dacarbazine-containing regimens [36]. Low-level, non-toxic doses of paclitaxel prevent DC precursors from becoming functionally tolerant. Moreover, there is evidence which suggests that low-toxicity doses of paclitaxel inhibit DCs and maintain the response to DC and lipopolysaccharide stimulation [37]. Mouse myeloma experiments have shown that injection of paclitaxel induces tumor-specific cytotoxic T-lymphocyte responses and prolongs tumor immunity. The apoptosis-inducing receptor CD95 (APO-1/Fas) plays a key role in apoptosis and is up-regulated with increasing RT25 [38].

Systemic immunological activity was measured by multiplex analysis and flow cytometry, and the response was positively correlated with higher tumor CD3+ infiltration (immunization score). This is characterized by a pre-existing systemic inflammatory state in which there is an increase in both selected chemokines and advanced B-cell differentiation, both of which are associated with poor prognosis [39]. Adoptive cellular immunotherapy (using DCs and cytokine-induced killer [CIK] cells) is a cancer treatment strategy in which tumor cells themselves or killer cells of allogeneic tumors are perfused. CIK cells have many immune cell properties. For example, the non-major histocompatibility complex of NK cells and the strong antitumor activity of T lymphocytes grant these cells the advantages of rapid proliferation, high killing activity and broad spectrum of tumor killing, and few side effects on bone marrow hematopoiesis. The interaction between DCs and CIK cells has a mutual promoting effect [40].

Paclitaxel inhibits cell mitosis and is a first-line chemotherapy drug. Paclitaxel chemotherapy can increase the rate of apoptosis in tumor cells, release tumor antigens, and enhance the phagocytosis of antigen-presenting cells (APCs). APCs are activated to release more pro-inflammatory cytokines, thereby promoting the cross-presentation of APCs with tumor antigens. DC immunotherapy is used to inject the patient’s own immune cells back into the patient after activation, modification, and proliferation in vitro, thereby inducing a specific or non-specific immune response, killing tumor cells. DCs are a class of heterogeneous cells that play an important regulatory role in cellular and humoral immunity. These cells have high killing activity and make up 1–5% of peripheral human peripheral blood lymphocytes. It has been clinically confirmed that extensive amplification of DCs results in significant tumor-killing and virus-eliminating effects.

Paclitaxel has been shown to inhibit the function of Tregs and reverse the immune escape of tumors. CIK or DC-CIK adoptive immunotherapy can kill tumor cells. Therefore, paclitaxel combined with immunotherapy could increase the efficacy of treatment. Clinically, paclitaxel combined therapy has been used to treat breast cancer, NSCLC, ovarian cancer, and other malignant tumors. Clinical studies using paclitaxel–carboplatin–bevacizumab in concert to treat lung cancer are in stage IIIB or stage IV [41]. One study aimed to evaluate the efficacy and toxicity of liposomal paclitaxel and carboplatin combined with radiotherapy for locally advanced lung squamous cell carcinoma (LSCC) [42].

In human cancers, tyrosine kinases of the epidermal growth factor receptor (EGFR) family are frequently mutated [43]. Tyrosine kinase inhibitors (TKIs) of EGFRs have been used as the standard first-line therapy for patients with advanced NSCLC, but the development of secondary resistance has led to treatment failure [44]. Paclitaxel is insoluble in water (less than 0.03 mg/ml), and due to this, the development of the drug was suspended for more than a decade [45]. Molecular-targeted therapy has become an attractive anticancer approach. Liposomal paclitaxel and carboplatin combined with radiotherapy have been shown to have significant antitumor effects on LSCC and controllable toxicity. These results indicate that liposomal paclitaxel-based chemoradiotherapy is a safe treatment for locally advanced LSCC, especially in allergic diseases. Studies of the combination of the EGFR T790 M-targeting inhibitor AZD929 and paclitaxel in the treatment of lung cancer have shown that the two have strong synergy, both in cell cultures and in vivo, without additional toxicity [46–49]. Mark and colleagues demonstrated that albumin-bound paclitaxel is a safe and effective therapeutic agent for NSCLC [50, 51]. Julide and coworkers compared two taxanes in the second-line treatment of NSCLC and found that there was no significant difference in survival, treatment response, or side effects between the two [52].

Molecular biology studies have shown that the NF-kappa-B inhibitor (IKB-α/NF-κB/Bcl-2) and EGFR/Akt pathways work synergistically. To investigate the efficacy and safety of bevacizumab combined with CP in the treatment of advanced NSCLC [53–55], the combination of CP and bevacizumab can be compared to PC alone. However, this combination may result in a higher toxicity profile. Therefore, the benefits and risks should be considered before making a treatment decision. Little is yet known about this, as immunotherapy is still in the research stage. The immune effect of treatment on cancer patients is not straightforward, and the “window period” of immunotherapy combined with chemotherapy also remains unclear. One study investigated the immunogenicity of CP-induced apoptosis in ovarian cancer cells, the immunological aspects of ovarian cancer chemotherapy in patients, and the cytotoxic T lymphocyte (CTL) response of tumor antigens to CD8(+) T cells in the window phase [56]. Paclitaxel acts by interfering with normal microtubule breaks during cell division. The ratio of CD3+ T cells, CD4+ T cells, and CD4+ cells in S1, S2, and S3B cells did not change significantly with the ratio of CD0+ cells. The ratio of IFN-γ production in S2 and Tc1 cells and the ratio of TC1 cells to TC2 cells increased in S2 upon treatment. The study also found that CD4 + CD45 RO+ and CD8 + CD45 Ro + memory T cells were significantly increased in S2 compared to CD0.

Increases in the number of memory T cells may provide an opportunity for developing long-term immune memory, and providing protection for recurrence and metastasis after chemotherapy in patients with ovarian cancer. Tumors actively recruit and induce Tregs to block innate and adaptive immune initiation, its effects, and the memory response. Paclitaxel and carboplatin have high immunogenicity and induce apoptosis in ovarian cancer cells. The immunity of patients with advanced ovarian cancer is impaired. After chemotherapy, the immune system recombines, thus providing a unique opportunity to use therapeutic interventions that modulate the reactivity of tumors to their antigens. In the chemotherapy cycle of CP in patients with advanced ovarian cancer, the 12th to 14th day after chemotherapy may be an ideal time to implement immunotherapy.

### Summary and outlook

Paclitaxel is the most powerful natural product available to treat cancer. Today, chemical semi-synthesis and artificial cultivation of the yew are the main sources of paclitaxel. Increasing the production of paclitaxel is an urgent need, and comprehensive research among various disciplines is necessary. Although total chemical synthesis is a very effective means to produce paclitaxel, the complicated synthetic route of paclitaxel and the high cost hinder its industrial production. Plant cell culture is a more promising and sustainable way to produce paclitaxel, but the costs of production are even higher and the yields are less predictable. Although endophytic fungi have been found to have great prospects for paclitaxel production, the biosynthetic pathway of paclitaxel is still unclear and the yield is not high.

With the development of biosynthesis technology, it is possible that microbial cells will be used to produce paclitaxel in the future. In this case, it would be necessary to use various breeding methods. At present, there are many studies detailing the use of mutagenesis in obtaining high-yield, paclitaxel-producing strains. Molecular breeding and metabolic regulation breeding techniques are also involved. More research is needed to clearly characterize the biosynthetic pathway of paclitaxel in microorganisms. Paclitaxel can affect the outcome of immunotherapy by various mechanisms of action on immune cells, and it also plays a role as an immunomodulator. However, the tumor immune process is complicated and cancer is difficult to cure. The function of paclitaxel in tumor immunotherapy interventions needs to be further studied.

## Data Availability

Not applicable.

## Abbreviations

AIDS: Acquired immunodeficiency syndrome
APC: Antigen-presenting cell
CIK: Cytokine-induced killer
CMCs: Cambial meristematic cells
CP: Carboplatin and paclitaxel
CTL: Cytotoxic T lymphocyte
DC: Dendritic cell
DCCP: Dendritic cell vaccine in combination with carboplatin and paclitaxel chemotherapy
DDCs: Dedifferentiated cells
EGFR: Epidermal growth factor receptor
HMGB1: High-mobility group box one
IL: Interleukin
LSCC: Lung squamous cell carcinoma
NF-kappa-B: The nuclear factor ‘kappa-light-chain-enhancer’ of activated B cells
NK: Natural killer
NSCLC: Non-small cell lung cancer
PD-L2: Programmed death receptor-ligand 2
TKI: Tyrosine kinase inhibitor
Treg: Regulatory T cell
TS: Taxadiene synthase
